# Adsorption Analysis of Lactoferrin to Titanium, Stainless Steel, Zirconia, and Polymethyl Methacrylate Using the Quartz Crystal Microbalance Method

**DOI:** 10.1155/2016/3961286

**Published:** 2016-02-21

**Authors:** Eiji Yoshida, Tohru Hayakawa

**Affiliations:** Department of Dental Engineering, Tsurumi University School of Dental Medicine, 2-1-3 Tsurumi, Tsurumi-ku, Yokohama 230-8501, Japan

## Abstract

It is postulated that biofilm formation in the oral cavity causes some oral diseases. Lactoferrin is an antibacterial protein in saliva and an important defense factor against biofilm development. We analyzed the adsorbed amount of lactoferrin and the dissociation constant (*K*_*d*_) of lactoferrin to the surface of different dental materials using an equilibrium analysis technique in a 27 MHz quartz crystal microbalance (QCM) measurement. Four different materials, titanium (Ti), stainless steel (SUS), zirconia (ZrO_2_) and polymethyl methacrylate (PMMA), were evaluated. These materials were coated onto QCM sensors and the surfaces characterized by atomic force microscopic observation, measurements of surface roughness, contact angles of water, and zeta potential. QCM measurements revealed that Ti and SUS showed a greater amount of lactoferrin adsorption than ZrO_2_ and PMMA. Surface roughness and zeta potential influenced the lactoferrin adsorption. On the contrary, the* K*_*d*_ value analysis indicated that the adsorbed lactoferrin bound less tightly to the Ti and SUS surfaces than to the ZrO_2_ and PMMA surfaces. The hydrophobic interaction between lactoferrin and ZrO_2_ and PMMA is presumed to participate in better binding of lactoferrin to ZrO_2_ and PMMA surfaces. It was revealed that lactoferrin adsorption behavior was influenced by the characteristics of the material surface.

## 1. Introduction

It is now postulated that biofilm formation in the oral cavity causes oral diseases such as dental caries, periodontal diseases, denture-induced stomatitis, and peri-implantitis [1]. Biofilm includes a complex of attached bacterial and salivary macromolecules and forms on not only the teeth and mucosal surface but also the surface of metal or ceramic prostheses, orthodontic brackets, resin restoratives, and titanium implants [2, 3]. However, some defense proteins such as lactoferrin, lysozyme, and peroxidase, which are present in innate human salivary, are known to exert wide antimicrobial activity against a number of bacterial, viral, and fungal pathogens in vitro [4]. Lactoferrin in saliva represents an important defense factor against bacterial injuries including bacterial growth, biofilm development, iron overload, reactive oxygen formation, and inflammatory processes. The first function attributed to the antibacterial activity of lactoferrin was to bind iron, which is necessary for bacterial growth and survival [5]. Lactoferrin is used in oral health care products such as dentifrices, mouth-rinses, moisturizing gels, and chewing gums [6]. Some have tried to modify the surface of dental materials by applying a lactoferrin coating. Nagano-Takebe et al. reported that a lactoferrin coating on a titanium surface inhibited bacterial adhesion and exhibited bactericidal effects [7]. Furthermore, lactoferrin adsorbed to contact lenses (polyHEMA or silicone hydrogel) had previously been shown to kill Gram-negative bacteria [8].

The initial stage of biofilm formation is the adhesion of salivary proteins. The surface of restorative and/or prosthodontic dental materials exposed to the oral environment is covered by salivary proteins. Few studies have reported the adsorption of salivary proteins on the surface of artificial dental materials. Previously, we investigated the initial adsorption of salivary proteins onto different materials, that is, gold, silica, and titanium, by using the quartz crystal microbalance (QCM) method and identified the differences in the adsorption behaviors of salivary proteins [9]. The adsorbed amount of lactoferrin on a silica surface was significantly lower than that of gold and titanium surfaces. The QCM technique can detect the adsorption of protein onto a materials surface by measuring the difference in the oscillating frequency of the quartz cell [10].

In the present study, we analyzed the dissociation constant (*K*_*d*_) of lactoferrin to the surface of different dental materials, as well as the adsorbed amount of lactoferrin, by using the equilibrium analysis technique in a QCM measurement.* K*_*d*_ indicates the strength of the affinity between two molecules at the equilibrium state. The surfaces of different dental material, titanium, stainless steel, zirconium, and PMMA, were evaluated. These materials were coated onto QCM sensors. Some surface characteristics such as surface roughness, hydrophilicity, and the zeta potential of different dental materials were also measured.

## 2. Materials and Methods

### 2.1. QCM Apparatus and Sensors

A 27 MHz QCM (AT cut shear mode, AFFINIX QN*μ*, Initium Co., Ltd., Tokyo, Japan) with a 500 *μ*L cell was used. The temperature control system and stirring bar were equipped. The temperature was maintained at 25  ±  1°C and the solution in the cells was stirred during the measurements.

Titanium (Ti), stainless steel (SUS), zirconia (ZrO_2_), and polymethyl methacrylate (PMMA) sensors were used. The Ti, SUS, and ZrO_2_ sensor was prepared by the sputter coating of each material on an Au electrode. The deposit conditions of Ti, SUS, and ZrO_2_ sensor are shown in Table 1. Ti, SUS, or zirconium disks (Quartz 4N, ULVAC, Inc., Kanagawa, Japan) were used as a target, and the deposition of each material was performed using sputtering deposition equipment (CS200, ULVAC, Inc., Kanagawa, Japan). Ti or SUS sputtering was performed under argon gas, and, on the other hand, zirconium sputtering was under oxygen gas to deposit ZrO_2_. The PMMA sensor was prepared by spin coating on an Au electrode. 2% PMMA polymer (Wako Pure Chemical Industries, Ltd., Osaka Japan) in toluene was spin-coated at 4000 rpm for 2 minutes. Each sensor was irradiated by ultraviolet radiation (BioForce Nanosciences Holdings Inc., US) for 20 minutes before QCM measurement except for the PMMA sensor.

### 2.2. Atomic Force Microscope (AFM) Observation of Different Sensors

An AFM (Nanosurf Easyscan 2, Nanosurf, AG, Switzerland) observation identified the surface condition and surface roughness (Sa) of the different sensors. AFM images were captured in air by the tapping mode. Tapping mode silicon probes (Tap190AL-G, force contact 48 N/m Budget sensors, Bulgaria) with resonance frequencies of approximately 190 kHz were used for imaging. The AFM images were obtained in an area with 2 × 2 *μ*m^2^.

### 2.3. Contact Angle Measurements of Different Sensors

The contact angles of different sensor surfaces with respect to double-distilled water were measured using a contact angle meter (CA-P; Kyowa Interface Science Co., Ltd., Tokyo, Japan) after the ultraviolet irradiation of each sensor except for the PMMA sensor. The water drop volume was maintained at 2 *μ*L, and three measurements of 20 seconds each were made for each surface type. Measurements were performed at the same room temperature and humidity.

### 2.4. Apparent Zeta Potential Measurements of Different Sensors

*Apparent* zeta potentials for the solid surface of Ti, SUS, ZrO_2_, and PMMA plates (1 × 10 × 20 mm, Furuuchi Chemical Corp., Tokyo, Japan) were monitored with a SurPASS electro kinetic instrument (Anton Paar GmbH, Graz, Austria). Zeta potential indicates the electrical charging behaviors of the material surface. For determination of the zeta potential, electrolyte aqueous solution (1 mM NaCl) at a pH = 7.3, which was adjusted by 0.01 M HCl and 0.01 M NaOH, was used. An electrolyte solution passes through a thin slit channel formed by two identical sample surfaces. The streaming current and streaming potential resulting from the pressure-driven flow of an electrolyte were measured. Then zeta potential was calculated using the Helmholtz-Smoluchowski equation [11, 12].

### 2.5. QCM Measurements of Lactoferrin Adsorption by Equilibrium Analysis

Bovine milk lactoferrin (MW = 80 kDa, Wako Pure Chemical Industries, Ltd., Japan) was dissolved in a phosphate-buffered saline (PBS) solution (pH = 7.4) at a concentration of 0.1 and 0.5 mg/mL. The equilibrium analysis technique was employed to analyze the* K*_*d*_ (dissociation constant) of lactoferrin, as well as the adsorbed amounts, by 27 MHz QCM (AT cut shear mode, AFFINIX QN*μ*, Initium Co., Ltd., Tokyo, Japan). First, 500 *μ*L of PBS was added to the cell. After stabilization of the cell, a 0.1 mg/mL lactoferrin solution (0.5 *μ*L) was injected to the PBS solution in the cell successively four times every 20 minutes. At the fifth time of injection, a 0.5 mg/mL lactoferrin solution (0.5 *μ*L) was injected. The lactoferrin adsorption behavior by the equilibrium analysis technique was analyzed by AQUA software (Initium Co., Ltd., Tokyo, Japan). The maximum amount of adsorption saturation and* K*_*d*_ value was calculated by the Sauerbrey equation [13]. A frequency decrease of 1 Hz corresponds to 0.61 ± 0.1 ng cm^−2^ adsorption on the sensor in a 27 MHz QCM system [14].

### 2.6. Statistical Analyses

Significant differences were determined by one-way analysis of variance (ANOVA) using statistical analysis software (GraphPad Prism, GraphPad Software Inc., San Diego, CA, USA). Statistical significance was set at *p* < 0.05.

## 3. Results

### 3.1. Characterization of QCM Sensors

**Figure 1:**
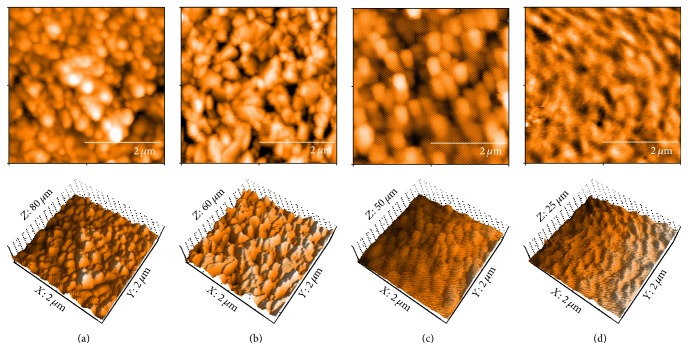
AFM images of Ti (a), SUS (b), ZrO_2_ (c), and PMMA (d) coated QCM sensors. All measurements were performed in tapping mode using aluminum reflex coating silicon long cantilever with a resonance frequency of approximately 190 kHz and force contact 48 N/m. The AFM images were obtained in an area with 2 × 2 *μ*m^2^.

No distinct differences in appearances were observed among the surfaces of the Ti, SUS, and ZrO_2_ sensors (Figure 1). Spherical particles with a diameter of 0.2~0.3 *μ*m were observed, while a wave-like structure was observed on the surface of PMMA. Sa values (average surface roughness in area) of the Ti, SUS, and ZrO_2_ sensors were not significantly different, although that of PMMA was significantly smaller than those of the others (Table 2). The contact angles of Ti and SUS were significantly lower than those of ZrO_2_ and PMMA; also, PMMA was significantly higher than those of other sensors (Table 3). The apparent zeta potential of Ti was significantly lower than those of the others, while that of ZrO_2_ was significantly higher compared to the others (Table 4). SUS and PMMA had almost the same zeta potential.

### 3.2. QCM Measurements

**Figure 2:**
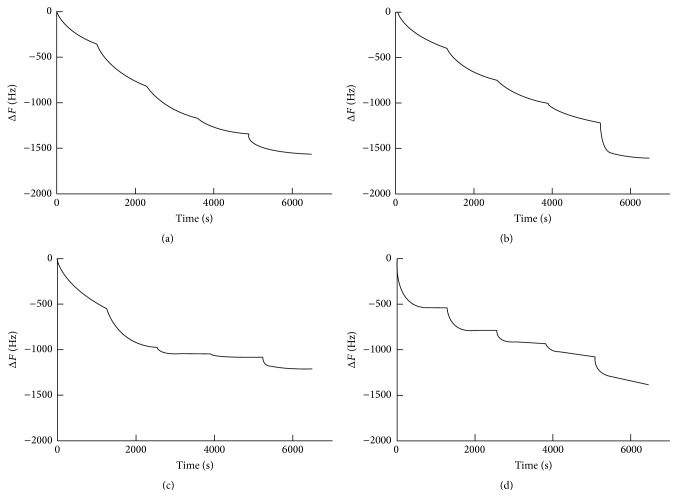
Frequency shift in a stepwise fashion after the injection of lactoferrin. (a) Ti, (b) SUS, (c) ZrO_2_, and (d) PMMA.

**Figure 3:**
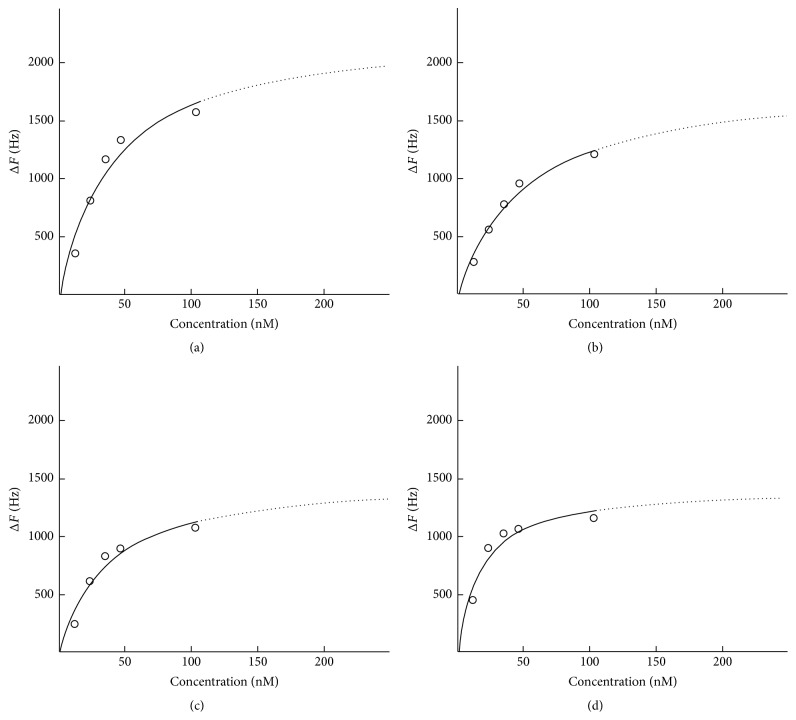
Langmuir isotherm nonlinear fitting of each sensor analyzed by AQUA. (a) Ti, (b) SUS, (c) ZrO_2_, and (d) PMMA.

**Figure 4:**
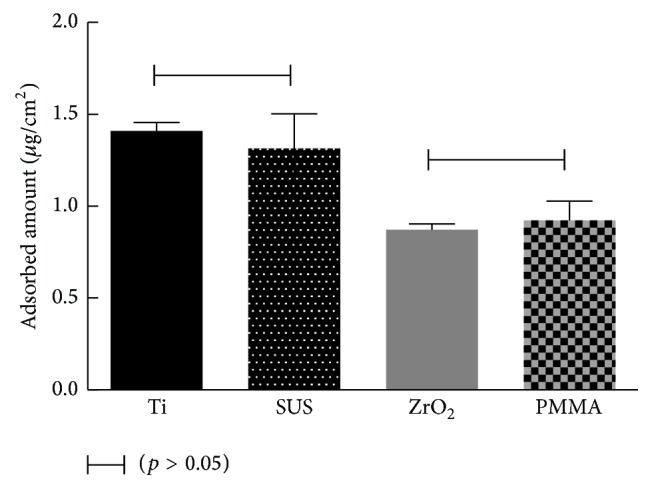
Adsorbed amount of lactoferrin to each sensor measured by an equilibrium analysis technique. Connected bar: no significant difference (*p* > 0.05).

**Figure 5:**
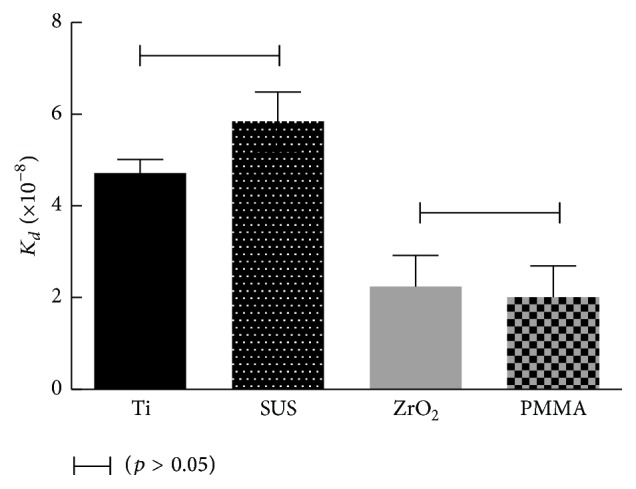
*K*_*d*_ values of each sensor resulting by nonlinear fitting analysis. Connected bar: no significant difference (*p* > 0.05).

The adsorption of lactoferrin to the different sensors was monitored by an equilibrium analysis technique. The frequency of each sensor decreased in a stepwise fashion after the injection of lactoferrin (Figure 2). The final injection caused a greater decrease in frequency due to the injection of lactoferrin at five times higher concentration, except for the ZrO_2_ sensor. The ZrO_2_ and PMMA sensors showed a greater degree of decrease in the frequency at the first and second injection of lactoferrin. Maximum adsorbed amounts and* K*_*d*_ values were obtained from the nonlinear fitting (Langmuir isotherm) between Δ*F* (decrease in the frequency) and the concentration of lactoferrin by equilibrium analysis (Figure 3). Adsorbed amounts of lactoferrin for the Ti and SUS sensors were significantly higher than those of the ZrO_2_ and PMMA sensors (Figure 4). There were no significant differences in the adsorbed amounts of lactoferrin between Ti and SUS and between the ZrO_2_ and PMMA sensors.* K*_*d*_ values for lactoferrin adsorption for the Ti and SUS sensors were also significantly higher than those of ZrO_2_ and PMMA (Figure 5). No significant differences were found in* K*_*d*_ values between Ti and SUS and between the ZrO_2_ and PMMA sensors.

## 4. Discussion

In the present study, we evaluated the adsorption of lactoferrin by using an equilibrium analysis technique in the QCM method. There are two analyses approaches for adsorption using the QCM method, namely, equilibrium analysis and kinetic analysis. In kinetic analysis, a certain concentration of adsorbed solution is injected once and the change of the frequency monitored. Adsorbed amounts and apparent reaction rate are observed for each measurement. In equilibrium analysis, a different concentration of adsorbed solution is injected over several time periods. Takakusagi et al. injected four different concentrations of camptothecin, 0.01, 0.05, 0.1, and 0.5 *μ*M, for the analysis of camptothecin binding to synthetic peptide using equilibrium analysis in the QCM method [15]. We preliminarily tried to inject the lactoferrin solution at different concentrations, for example, five injections of 0.5 mg/mL solution or 0.1 mg solution. In the present experiments, four injections of 0.1 mg/mL solution and a final injection of 0.5 mg/mL were employed because of an improvement in the nonlinear correlation between Δ*F* and the concentration of lactoferrin. We used 27 MHz of fundamental frequency quartz crystal, which means the quartz crystal is about 30 times more sensitive than the 5 MHz conventional quartz crystal microbalance.

As materials for lactoferrin adsorption, two metal materials, Ti and SUS, one ceramic, ZrO_2_, and one organic polymer material, PMMA, were used. These materials are used as dental implants, orthodontic wire, crowns and inlays or denture plates, and so forth in dental clinics. The Ti, SUS, and ZrO_2_ surfaces were prepared by sputter coating and the PMMA surface was prepared by spin coating. These surfaces were characterized by AFM observation, measurement of surface roughness, contact angles of water, and zeta potential.

The results obtained from the measurements of adsorbed amounts of lactoferrin and* K*_*d*_ values were classified into two groups by the types of sensor materials. Consequently, the Ti and SUS group showed significantly greater amounts of lactoferrin adsorption and* K*_*d*_ values than the ZrO_2_ and PMMA group.

The isoelectric point (PI) of lactoferrin was 8.2~8.9 and the electrical status positively charged at pH = 7.3 [16]. Electrostatic interaction between protein and titanium has been reported to be dominant in the protein adsorption [9, 17]. The four different surfaces tested in the present study were negatively charged by measuring zeta potential. It can be predicted that a larger amount of lactoferrin will be adsorbed onto a more negatively charged surface. Ti was shown to have a more negatively charged zeta potential and indeed a greater amount of lactoferrin adsorbed onto Ti probably due to this negative charge. The lower amount of lactoferrin adsorbed onto the ZrO_2_ surface may be due to this surfaces relatively small negative charge. Although SUS and PMMA have almost the same zeta potential, the adsorbed amount of lactoferrin to SUS was significantly greater than for PMMA. It is well known that the surface roughness of a material surface influences the adsorption of proteins [18]. A rougher surface provides greater amounts of protein adsorption. SUS has a more roughened surface than PMMA. It is possible that the larger roughness of the SUS surface allowed a larger amount of lactoferrin to adsorb compared to the PMMA surface. Hydrophobic or hydrophilic interaction is another candidate for controlling the adsorption behavior of proteins to a material surface [19]. In the present study, the more hydrophilic surfaces, Ti and SUS, provided greater amounts of lactoferrin adsorption.

*K*_*d*_ values mean the affinity of lactoferrin to material surface. A smaller* K*_*d*_ value corresponds to a better affinity of lactoferrin to the material surface. The present results for* K*_*d*_ values revealed that the affinity of lactoferrin to ZrO_2_ and PMMA was greater than that of Ti and SUS. The ZrO_2_ and PMMA surfaces were more hydrophobic than those of Ti and SUS. For the adsorption of lactoferrin, a more hydrophilic surface provided a larger amount of adsorption. However, tighter binding of lactoferrin to a more hydrophobic surface was obtained. The reason for this controversial phenomenon was not clear, but it assumed that the hydrophobic interaction participated in the superior binding of lactoferrin to the ZrO_2_ and PMMA surfaces than to those of Ti and SUS. Moreover, adsorption and desorption rate contribute the* K*_*d*_ values. In this study desorption behavior was not monitored, and adsorption and desorption rate are evaluated by using a kinetic analysis as mentioned above. Detailed studies on the interaction between adsorbed lactoferrin and material surface including desorption behavior are needed by using a kinetic analysis in addition to equilibrium analysis.

## 5. Conclusions

The present study revealed that lactoferrin adsorption behavior was influenced by the characteristics of the material surface. Ti and SUS showed a greater amount of lactoferrin adsorption than ZrO_2_ and PMMA, but the adsorbed lactoferrin bound less tightly to the Ti and SUS surfaces than to ZrO_2_ and PMMA surfaces. The influence of other factors, such as conformational changes of lactoferrin and electrolyte used, will be further investigated. It is also necessary to consider how the adsorption of lactoferrin influences biofilm formation on different dental materials in the future.

## Figures and Tables

**Table 1:** Deposit conditions of Ti, SUS, and ZrO_2_ sensors.

Sensor	Target	Gas	Pressure (Pa)	Time (min)
Ti	99.99% pure Ti	Argon	0.2	30
SUS	SUS304	Argon	0.1	10
ZrO_2_	Zirconium	Oxygen	0.5	30

**Table 2:** Surface roughness of sensors (mean ± SD, *n* = 3).

Sensor	Surface roughness (Sa: 4 *µ*m^2^)
Ti	4.45 ± 0.50^a^
SUS	5.35 ± 0.51^a^
ZrO_2_	4.61 ± 0.25^a^
PMMA	1.30 ± 0.25^b^

The same small letters denote no significant differences (*p* > 0.05).

**Table 3:** Result of contact angle measurements (mean ± SD, *n* = 3).

Sensor	Contact angle (°)
Ti	13 ± 1.5^a^
SUS	9 ± 1.2^a^
ZrO_2_	32 ± 2.7^b^
PMMA	64 ± 1.5^c^

The same small letters denote no significant differences (*p* > 0.05).

**Table 4:** Measurements of apparent zeta potential (mean ± SD, *n* = 4).

Sensor	Zeta potential (mV)
Ti	−86.6 ± 5.4^a^
SUS	−64.8 ± 8.0^b^
ZrO_2_	−43.2 ± 3.3^c^
PMMA	−64.8 ± 4.0^b^

The same small letters denote no significant differences (*p* > 0.05).
